# Supplementary material for: Real-time tracking of coherent oscillations of electrons in a nanodevice by photo-assisted tunnelling
Source: Nat Commun. 2024 Feb 13;15:1316. doi: 10.1038/s41467-024-45564-w (PMC10864318; doi:10.1038/s41467-024-45564-w)
Supplement: Supplementary file 1 — Supplementary Information [file 41467_2024_45564_MOESM1_ESM.docx]

Supplementary Information for

**Real-Time Tracking of Coherent Oscillations of Electrons in a Nanodevice by Photo-assisted Tunnelling**

Yang Luo^1^, Frank Neubrech^1,2^, Alberto Martin-Jimenez^1^, Na Liu^1,2^, Klaus Kern^1,3^, Manish Garg^1,#^

^1^ Max Planck Institute for Solid State Research, Heisenbergstr. 1, 70569 Stuttgart, Germany

^2^ 2^nd^ Physics Institute, University of Stuttgart, Pfaffenwaldring 57, 70569 Stuttgart, Germany

^3^ Institut de Physique, Ecole Polytechnique Fédérale de Lausanne, 1015 Lausanne, Switzerland

^#^Author to whom correspondence should be addressed. [mgarg@fkf.mpg.de](mailto:mgarg@fkf.mpg.de)

**Section I. Non-collinear second-harmonic frequency-resolved optical gating (FROG) measurements**


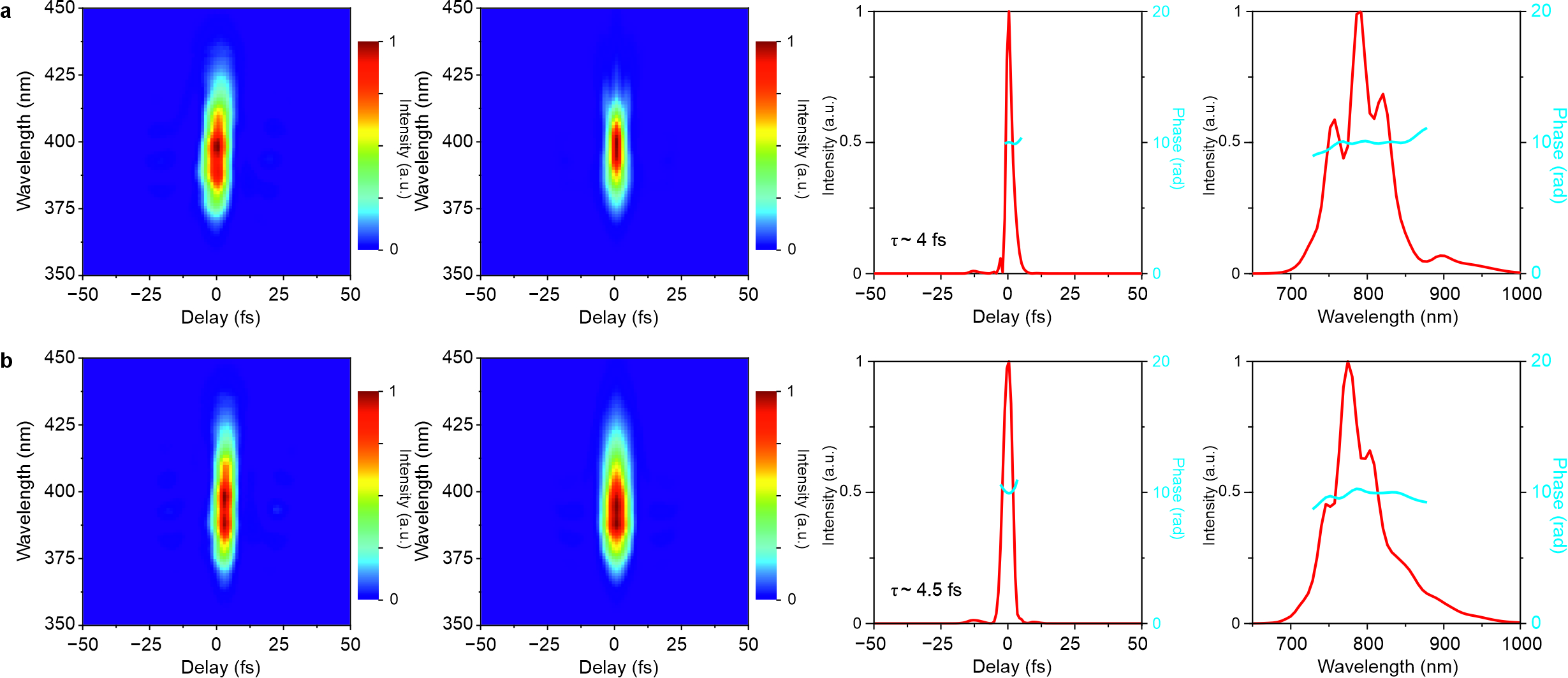


**Fig. S1 | FROG measurements and reconstructions for pulse-2 (a, the original laser beam) and pulse-1 (1^st^ order diffraction beam, b).** First Column: The experimental FROG traces. Second Column: The reconstructed FROG traces. Third Column: The temporal profiles of laser intensity (red-curve) and phase (blue-curve). Fourth Column: The spectral profiles of laser intensity (blue-curve) and phase (red-curve). The reduced bandwidth of the laser pulses as retrieved from the FROG measurements is due to the limited spectral phase matching bandwidth of the ~ 10 μm thick BBO crystal as used in the FROG set-up.

**Section II. Finite element simulations of a single bowtie**

Numerical simulations were performed using the commercial software COMSOL Multiphysics

based on a finite element method. A single bowtie structure is implemented by two identical but opposing isosceles triangles with a base of 250 nm and a height of 300 nm supported by a substrate. The opposing tips and edges of the bowties are modelled with filets (radius of 5 nm). The height of the bowtie is 30 nm and a junction size of 10 nm is exemplarily selected. The dielectric function of gold was taken from Johnson and Christy^1^ and the refractive index of the fused silica substrate was approximated with 1.5 in the spectral range of interest. A refined mesh size of at least 1 nm was used in a volume (50 nm x 50 nm x 50 nm) centered in the bowtie junction to map the fine features of the junction. Perfectly matched layers were placed around the simulation domain to completely absorb the waves leaving the domain. The spectral response and the 2D electrical field distributions of a single bowtie structure are numerically calculated using full field formulation and background field conditions.

Figure S2 shows the extinction cross section of a single bowtie with the abovementioned dimensions. The insets depict the electrical field distributions (taken at the half height of the bowtie) normalized to the background electrical field at the respective wavelengths. The polarization of the incident electrical field is parallel to the bowtie axis. Based on the field distributions we identify the peak at 1420 nm as the first order plasmonic mode and the peaks at 770 nm and 810 nm as a higher order excitation originating from the hybridization of the two opposing isosceles triangles. The double peak feature results from plasmonic excitations in the electrical connections and is not present for bowties without electrical connections (not shown). The shoulder at ~1150 nm has the same origin.


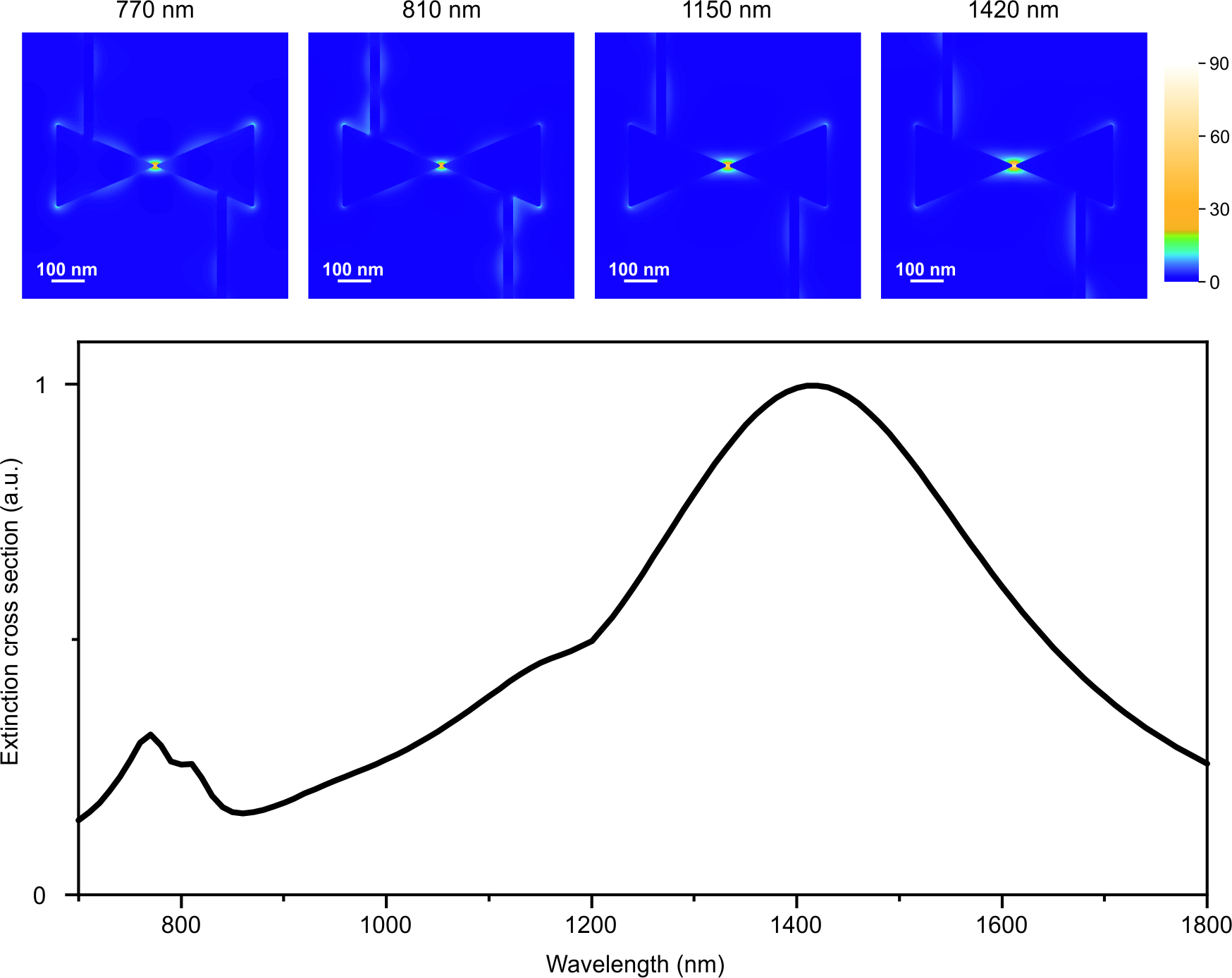


**Fig. S2 |** Bottom Panel: Simulated extinction cross section of a single bowtie as a function of the wavelength with dimensions given in the text. Top Panels: Distributions of the enhanced near fields at different wavelengths.

**Section III. Homodyne beating detection of the photo-assisted tunnelling current in Nanodevices**

An ultrashort CEP stable laser pulse produced in a mode-locked oscillator entails an underlying frequency comb, whose teeth are separated by the repetition rate (*f_r_*~ 80 MHz) of the oscillator. The frequency comb spans over the entire spectral bandwidth of the laser pulses, i.e. from 650 nm to 1050 nm, with the central wavelength of the spectrum being at ~ 810 nm. The electric field of this laser pulse in the frequency domain can be expressed as

$E_{1}(f_{1})=\sum_{n} \varepsilon(f_{n})\delta(f-f_{n})e^{ik.r}$, (1)

where *f_n_* = n*f_r_* , is the n^th^ multiple of the repetition rate of the laser pulses. $\delta(f-f_{n})$is the Dirac delta function describing the position of an individual tooth of the frequency comb, which are separated from each other by *f_r_* and $\varepsilon(f_{n})$ describes the spectral weight of each individual comb line in the laser spectrum. The central (or the carrier) frequency (*f*_1_) of our laser pulses is ~ 0.37$\times$10^15^ Hz (for a central wavelength of λ ~ 810 nm), implying that *n* ~ $\frac{f_{1}}{f_{r}}$ is approximately 4.6$\times$10^6^. This frequency comb is emitted in every shot from the oscillator at its repetition rate.

On interaction with the radio frequency (RF) wave in the acousto-optic-frequency-shifter (AOFS) being driven at the frequency of *f_r_* + *f_0_*, the ultrashort laser pulses undergo diffraction (Fig. 1d, main-text). The frequency comb of the 1^st^ order diffracted laser pulse (‘pulse-1’ in Fig. 1d) out of the AOFS is upshifted in the frequency by *f_r_* + *f_0_* with respect to the original laser pulse (‘pulse-2’ in Fig. 1d). The electric field of the first-order diffracted laser pulse from the AOFS can be expressed as;

$$E_{2}(f_{2})=\sum_{n} \varepsilon(f_{n}+f_{r}+f_{0})\delta(f-f_{n}-f_{r}-f_{0})e^{ik.r}$$

$E_{2}(f_{2})=\sum_{n} \varepsilon((n+1)f_{r}+f_{0})\delta(f-(n+1)f_{r}-f_{0})e^{ik.r}$ (2)

Since the order of the frequency comb lines *n* is much greater than 1 (*n* ~ 4.6$\times$10^6^), implying (*n*+1) ~ *n*, the above equation simplifies to

$$E_{2}(f_{2})=\sum_{n} \varepsilon(nf_{r}+f_{0})\delta(f-nf_{r}-f_{0})e^{ik.r}$$

$E_{2}(f_{2})=\sum_{n} \varepsilon(f_{n}+f_{0})\delta(f-f_{n}-f_{0})e^{ik.r}$ (3)

Thus, the offset between the carrier frequencies of the 1^st^ and 0^th^ order diffracted laser pulses is only ~ *f*_0_. The excited collective electron oscillations in the junction will produce a local electric field, which can be expressed as a convolution of the incident laser field *E_i_*(*t*) and the optical response function (*R*(*t*)) of the nanoantennas; $E_{Li}(t)\propto E_{i}(t)*R(t)$, where the subscripts, *i* = 1, 2 denote the two different laser pulses (pulse-1 and pulse-2, in Fig. 1d). The net electric field generated by the combination of the two pulses with a delay *τ* between them at the nanoantenna junction, in the time domain can be written as:

$E(t)=E_{L1}(t)\exp(-if_{1}t)+E_{L2}(t-\tau)\exp(-i(f_{1}+f_{0})(t-\tau))+c.c$ (4)

where *f*_1_ is the carrier frequency of the original beam (pulse-2 in Fig. 1d). The total polarization response (linear as well as nonlinear) induced in the nanoantenna junction owing to its interaction with the ultrashort pulses can be expressed as;

$P(t)\propto\chi^{(1)}E(t)+\chi^{(2)}E(t)^{2}+\chi^{(3)}E(t)^{3}+...$ (5)

where $\chi^{(1)}$, $\chi^{(2)}$and $\chi^{(3)}$are linear, second and third order optical susceptibilities, respectively.

Photo-assisted tunnelling current (*I*_1_*^T^*) generated in the nanoantenna junction due to the linear polarization response (one-photon absorption) of the nanoantennas will be proportional to the square of the net first-order polarization response induced by the two laser pulses.

$$I_{1}^{T}(t)\propto\chi_{1}^{2}E(t)^{2}$$

$I_{1}^{T}(t)\propto\chi_{1}^{2}\left\{\begin{matrix} E_{L1}^{2}(t)\exp(-2if_{1}t)+E_{L2}^{2}(t-\tau)\exp(-2i(f_{1}+f_{0})(t-\tau))+... \\ ...+E_{L1}(t)E_{L2}(t-\tau)\exp(-i(f_{0}t-(f_{1}+f_{0})\tau)))+c.c. \end{matrix}\right\}$ (6)

Here, most of the components of the tunnelling current due to the linear polarization response come at very high frequencies, such as 2*f_1_* and 2(*f_1_ + f_0_*), which is mixed with the tunnelling current signal at 0 Hz in the nanoantenna junction, thus cannot be measured by lock-in detection owing to the limited bandwidth of the high gain current amplifier. The cross-terms in the above equation, arising due to interference of the plasmon oscillations induced by the two frequency-shifted laser pulses in the nanoantenna junction contribute to the generation of the photocurrent, which oscillates at the small offset frequency of *f_0_* (< 1 kHz) in the nanoantenna junction,

$I_{1}^{T}(t)|_{f_{0}}\propto\chi_{1}^{2}\left|E_{L1}(t)E_{L2}(t-\tau)\right|\cos(f_{0}t-(f_{1}+f_{0})\tau)$， (7)

which can be measured in the experiments.

In the above analysis we have only considered the linear phase terms of the laser pulses, which is the group delay i.e. *f_1_t* and (*f_1_+f_0_)t*, where *f_1_* = *nf_r_*. If we consider all the phase terms of the laser pulses, the electron tunnelling current can be expressed as: , where *ϕ_1_* and *ϕ_2_* are the complete temporal phases of the two laser pulses.

The phase terms for the two laser pulses can be expanded by Taylor’s series with all linear and nonlinear phase terms and

The zero phase is the CEP of the laser pulse, and are the group delays of the laser pulses, and . and the corresponding group delay dispersions (GDD), and so on. Hence,

(8)

The dispersion of the two laser pulses, i.e. GDD and higher order phases are identical in our experiment, $\approx$, $\approx$…. Thus, the polarization term at a fixed delay of *τ* between the two pulses can be expressed as;

(9)

Therefore, measuring the linear polarization induced tunneling current arising due to one-photon excitations in the nanoantenna junction as a function of the delay between the two pulses at their carrier offset frequency (*f_0_*) enables complete temporal characterization of the laser pulses.

At *τ* = 0 fs delay between the two pulses (pulse-1 and pulse-2), the above equation imitates the photo-assisted tunnelling current generated by the polarization response induced by a single laser pulse coming at the repetition rate of the small offset frequency of *f_0_* at the nanoantenna junction,

$I_{1}^{T}(t)|_{f_{0}}\propto\chi_{1}^{2}(E_{L1}(t)^{2}+E_{L2}(t)^{2}+2\left|E_{L1}(t)E_{L2}(t)\right|\cos(f_{0}t))$ (10)

The electric field strengths of the pulse-1 and pulse-2 in our experiments are identical, $E_{L1}\approx E_{L2}\approx\frac{E_{0}}{2}$, hence simplifying the above equation to;

$I_{1}^{T}(t)||_{\tau=0}\propto\frac{1}{4}\left|\chi^{(1)}E_{0}(t)\right|^{2}(1+\cos(f_{0}t))$ (11)

In the case of a higher order nonlinear interaction of the laser pulses with the nanoantenna junction, the photo-assisted tunnelling current produced in the nanoantenna junction will be proportional to the multiple power of the corresponding terms in the total polarization response. For example, the photo-assisted tunnelling current due to a coherent two-photon absorption or the 2^nd^ order nonlinear polarization response in the nanoantenna junction will be;

$$I_{2}^{T}(t)\propto\left\{\chi^{(2)}E_{0}(t)^{2}\right\}^{2}$$

$I_{2}^{T}(t)|_{\tau=0}\propto\frac{1}{4}\left|\chi^{(2)}E_{0}(t)^{2}\right|^{2}(1+\cos(f_{0}t))^{2}=\frac{1}{8}\left|\chi^{(2)}E_{0}(t)^{2}\right|^{2}(3+4\cos(f_{0}t)+\cos(2f_{0}t))$ (12)

Similarly, the third-order nonlinear response would generate a photo-assisted tunnelling current as given by;

$$I_{3}^{T}(t)\propto\left\{\chi^{(3)}E(t)^{3}\right\}^{2}$$

$$I_{3}^{T}(t)|_{\tau=0}\propto\frac{1}{8}\left|\chi^{(3)}E_{0}(t)^{3}\right|^{2}(1+\cos(f_{0}t))^{3}=\frac{1}{8}\left|\chi^{(3)}E_{0}(t)^{3}\right|^{2}(1+\cos^{3}(f_{0}t)+3\cos^{2}(f_{0}t)+3\cos(f_{0}t))$$

$I_{3}^{T}(t)|_{\tau=0}\propto\frac{1}{8}\left|\chi^{(3)}E_{0}(t)^{3}\right|^{2}(1+\frac{\cos(3f_{0}t)+3\cos(f_{0}t)}{4}+3\frac{1+\cos(2f_{0}t)}{2}+3\cos(f_{0}t))$ (13)

Measurement of the time-resolved photo-assisted tunnelling current as a function of the delay between pulse-1 and pulse-2 at the lock-in frequency of 2*f*_0_ enables sampling of the 2^nd^ order polarization response (or the 2^nd^ order nonlinear electron oscillations) of the nanoantennas to the ultrashort laser pulses; $I_{2}^{T}(t)|_{2f_{0}}=\frac{1}{8}\left|\chi^{(2)}E_{L1}(t)E_{L2}(t-\tau)\right|^{2}\cos(2f_{0}t-2(f_{1}+f_{0})\tau)$. Likewise, time-resolved measurement at 3*f*_0_ frequency in the lock-in detection enables sampling of the 3^rd^ order nonlinear polarization response;

$I_{3}^{T}(t)|_{3f_{0}}=\frac{1}{8}\left|\chi^{(3)}E_{1}(t)E_{2}(t-\tau)\right|^{3}\cos(3f_{0}t-3(f_{1}+f_{0})\tau)$. The oscillation period of the second and third-order nonlinear electron oscillations will be one-half (~1.4 fs) and one-third (~0.9 fs) of the local plasmon oscillations (~2.7 fs), respectively. In the current experiments using laser power up to 220 pJ, the contributions of third-order nonlinear are usually weak.

In the intensity scaling experiment shown in Fig. 3e (main-text), measurement of photo-assisted tunnelling current at the lock-in frequency of *f_0_* at the zero delay between pulse-1 and pulse-2 would contain tunnelling currents arising mainly from the linear and second polarization responses, as can be understood from Eqn. (11)-(13).

$I_{f_{0}}^{T}(t)|_{\tau=0}\propto\frac{1}{4}\left|\chi^{(1)}E_{0}(t)\right|^{2}(\cos(f_{0}t))+\frac{1}{8}\left|\chi^{(2)}E_{0}(t)^{2}\right|^{2}(4\cos(f_{0}t))$ (14)

However, all the individual terms in the above equation arising due to different orders of the optical response follow completely different scaling laws with respect to the increasing field strengths of the incident laser pulses. Scaling of the photo-assisted tunnelling current in Fig. 3e for the lock-in frequency of *f_0_* shows a switching from the slope of one to the slope of two, indicating the presence of only linear response at the lower intensity of the laser pulses and presence of both linear as well as second order responses in the nanoantenna junction at higher intensity of the laser pulses. Third-order response, which would contribute to a slope of three in Fig. 3e are not present for the intensities of the laser pulses used in this experiment. The intensity of the laser pulses was intentionally kept below ~220 pJ in order to avoid irreversible physical damage of the nanoantennas, which occurs at higher intensity of the laser pulses. Nevertheless, at a higher intensity (~ 300 pJ) of the laser pulses, third-order nonlinear response can be measured as shown in Fig. 3b (main text).

In the measurement shown in Fig. 3e (main-text), photo-assisted tunnelling current at the lock-in frequency of 2*f_0_* can only arise due to the presence of the second-order response in the nanoantenna junction, as it shows a purely quadratic scaling in the experiment (Fig. 3e, main-text).

$I_{2f_{0}}^{T}(t)|_{\tau=0}\propto\frac{1}{8}\left|\chi^{(2)}E_{0}(t)^{2}\right|^{2}(\cos(2f_{0}t))$ (15)

Measurement of the amplitude of the photocurrent at the 2*f_0_* frequency enables a direct access to the contribution of second-order nonlinear response in the photocurrent signal measured at the lock-in frequency of *f_0_* in Eqn. (12). At lower incident intensities of the laser pulse, i.e. below 100 pJ in Fig. 3e (main-text), only linear response can be excited in the junction of the nanoantennas, as the signal measured at the 2*f*_0_ frequency is below the noise level and the slope of the scaling curve is one. Only when the signal at 2*f_0_* frequency start to emerge, i.e. above 100 pJ, the second order nonlinear response are generated in the nanojunction and this is when the slope of the scaling curve in the *f_0_* signal gradually changes its value from one to two. Therefore, by recording the homodyne beating signal at *f_0_* and its harmonic frequencies (2*f*_0_ and higher), we demonstrate a direct identification of contributions of 1^st^ and 2^nd^ order light-induced polarizations (electron oscillations) induced in the nanoantenna junction in the measured photocurrents in the nanodevice.

**Section IV. Comparison of the experimentally measured plasmon oscillations with the simulations**


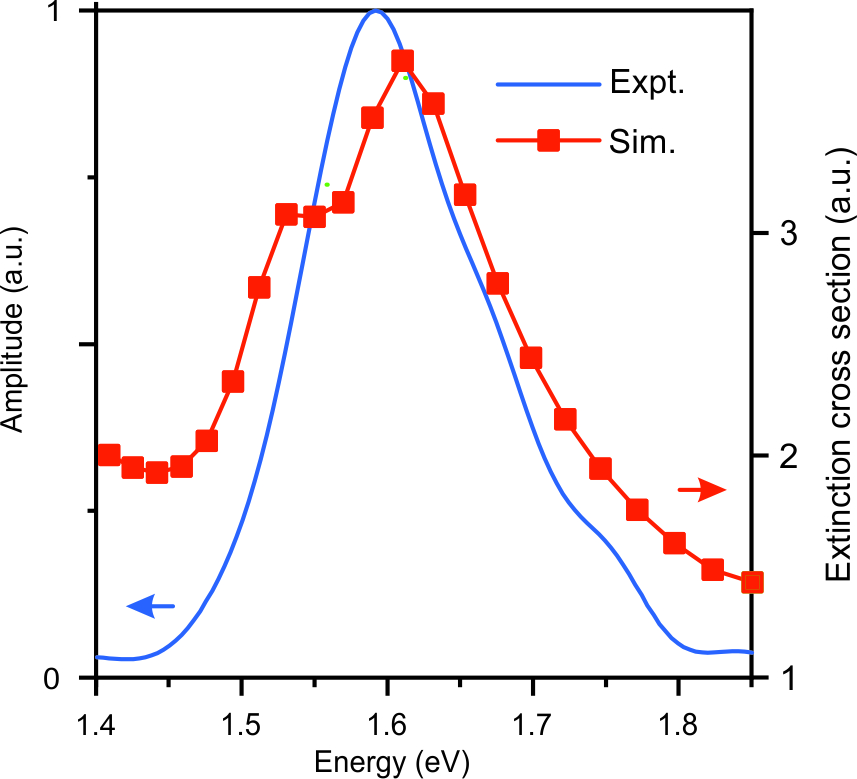


**Fig. S3 |** Comparison of the spectra of the experimentally measured and calculated plasmonic response of the nanoantenna junction. The blue curve shows the spectrum of the plasmonic response of the nanoantennas as measured in the experiments. The red curve shows the spectral shape of the plasmonic response as evaluated from the finite element simulations


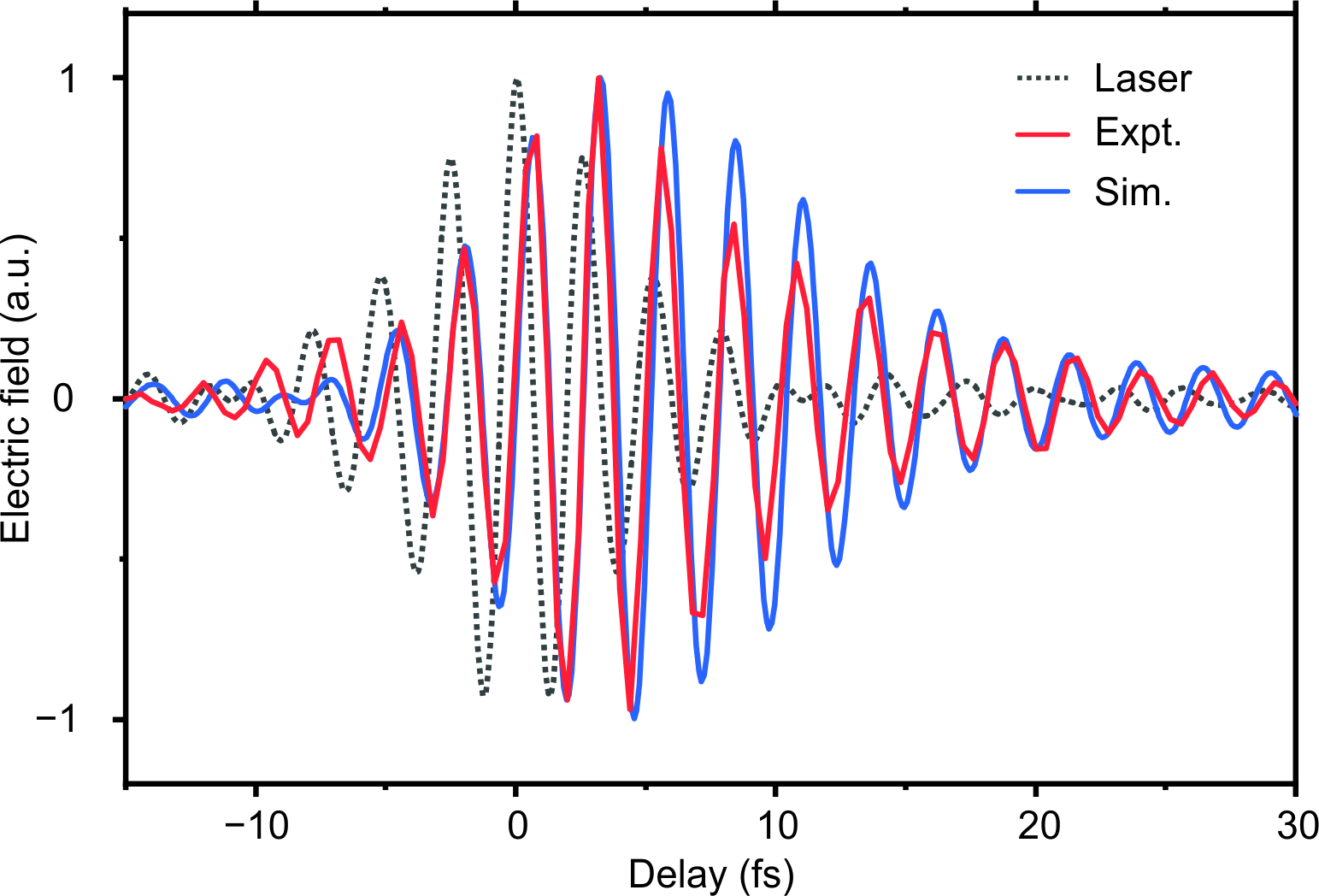


**Fig. S4 |** Comparison of the experimentally measured plasmon oscillations (the blue curve) with the electric field of the driving laser pulse (the dashed black curve) and the calculated plasmonic field (the red curve). The plasmon oscillations were measured by recording the variation of the laser-induced photocurrent as a function of the delay between pulse-1 and pulse-2 laser pulses of slightly different carrier frequencies. Its delay is shifted by ~ 3 fs to the positive side to allow a direct comparison with the calculated plasmonic field.


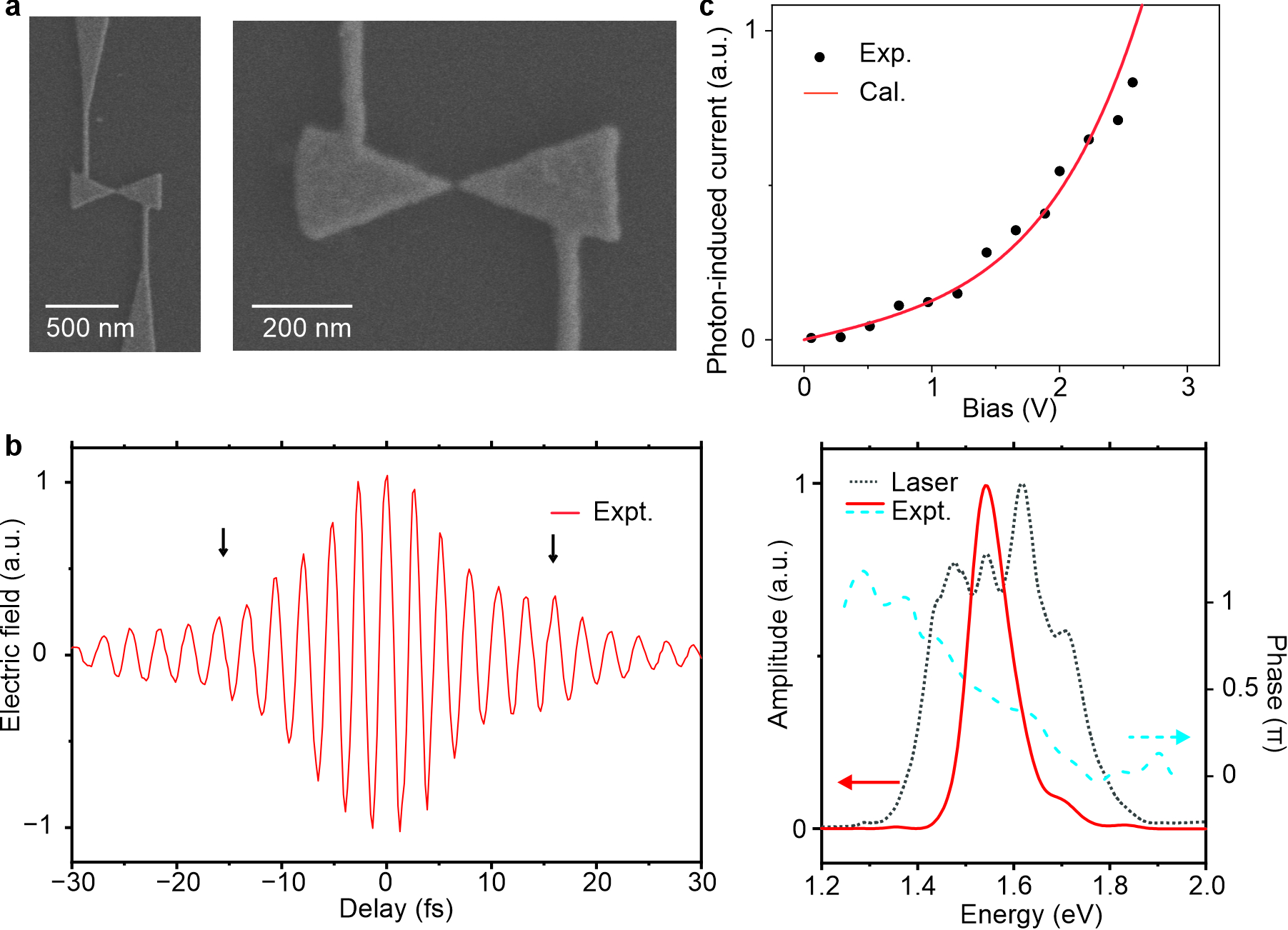


**Fig. S5 | Real-time tracking of coherent collective electron oscillations in a single bowtie nanoantenna. a,** Scanning electron microscope (SEM) images of single bowtie nanoantenna. **b,** Left-panel: Variation of the laser-induced photocurrent as a function of the delay between pulse-1 and pulse-2 laser pulses of slightly different carrier frequencies. The applied DC bias in the nanoantenna junction is 3 V, and the incident laser pulse energy is 100 pJ. Right-panel: The spectrum and phase of experimentally measured local plasmon oscillations in the nanoantnenna junction. The dotted black-curve shows the spectrum of the incident laser pulses on the nanoantenna junction. **c,** Variation of the photocurrent in the nanoantenna junction as a function of the applied DC bias. The red curve shows the calculated electron tunnelling probability considering only single-photon excitation with a junction gap (tunneling gap) of 0.7 nm.


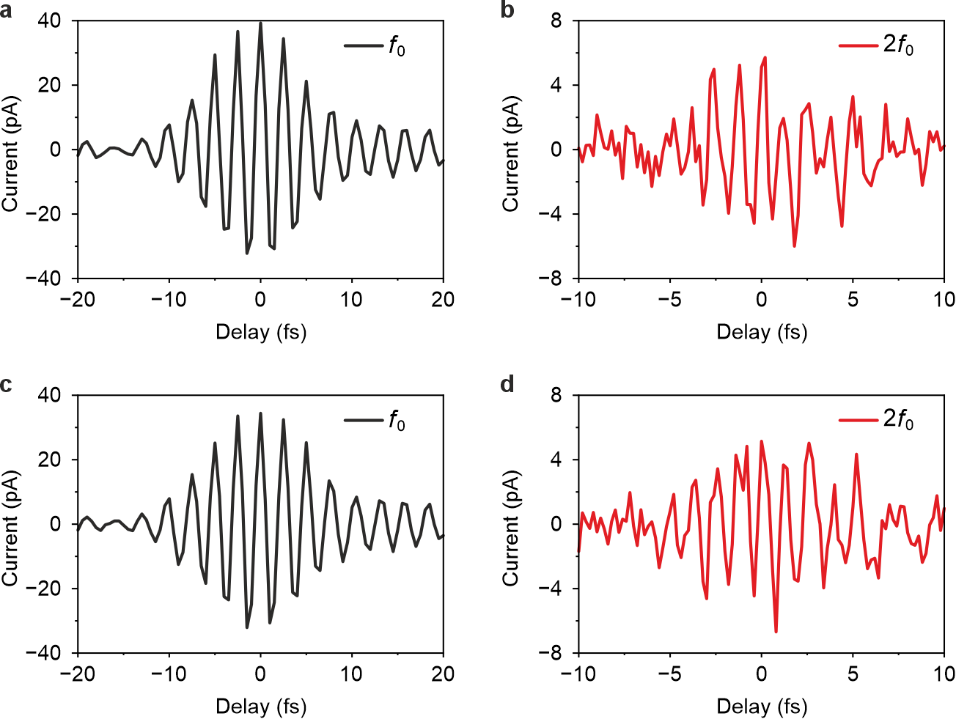


**Fig. S6 | Reproducibility of the intensity dependence measurements. a, b,** Variation of the laser-induced photocurrent as a function of the delay between pulse-1 and pulse-2 laser pulses measured at the lock-in frequency of *f_0_* (**a**) and 2*f_0_* (**b**) before starting the intensity dependence measurements shown in Fig. 3 of the main-text. **c, d,** Variation of the laser-induced photocurrent as a function of the delay between pulse-1 and pulse-2 laser pulses measured at the lock-in frequency of *f_0_* (**c**) and 2*f_0_* (**d**) after finishing the intensity dependence measurements shown in Fig. 3 of the main-text. The pulse energy of the laser pulses was set at ~ 200 pJ and the bias in the nanoantenna junction is 3.0 V.


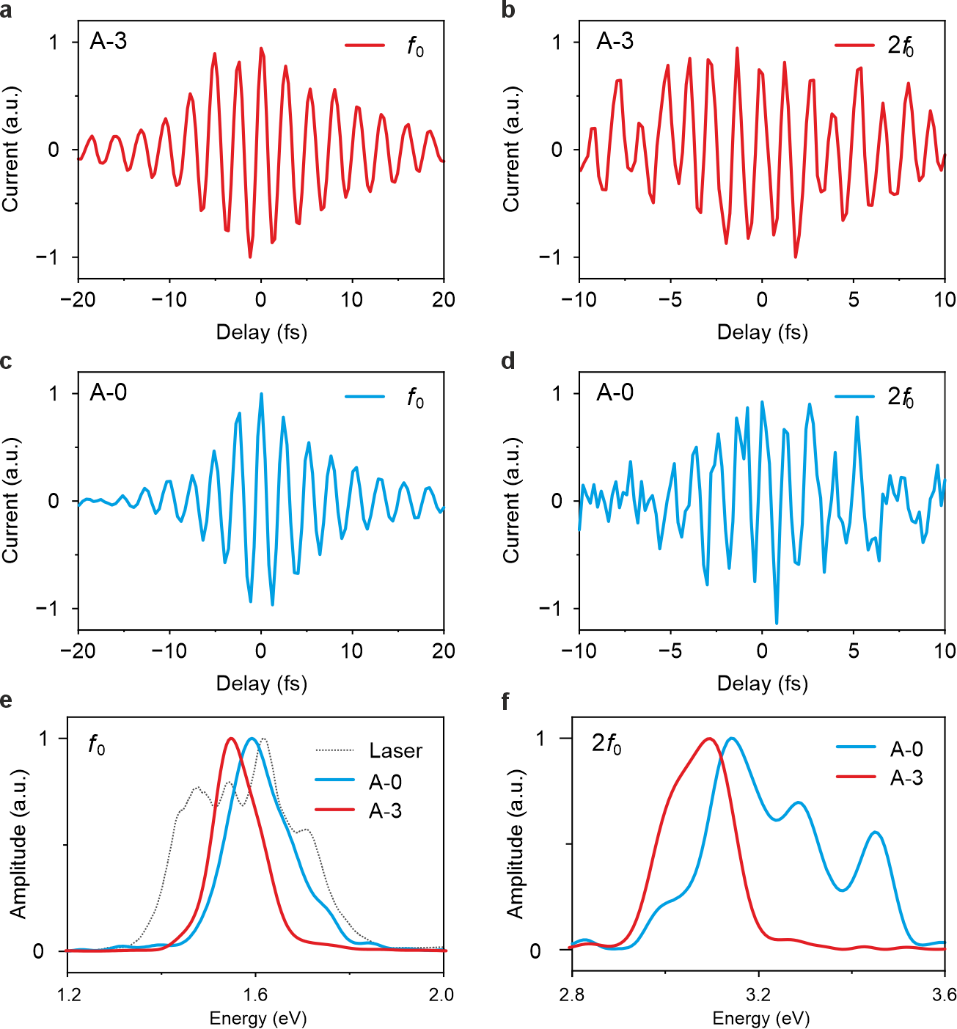


**Fig. S7 | Plasmon and nonlinear electron oscillations for different nanodevice geometry. a, b,** The variation of the linear (**a**) and nonlinear (**b**) laser-induced photocurrents measured as a function of the delay between pulse-1 and pulse-2 pulses for the nanodevice ‘A-3’ designed with a targeted gap size of −5 nm. The pulse energy of the laser pulses was set at ~ 200 pJ and the bias in the nanoantenna junction was 3.0 V. **c, d,** The temporal variation of the linear (**c**) and nonlinear (**d**) laser-induced photocurrents for a nanodevice ‘A-0’ (reproduced from Fig. 2a and Fig. 3a of the main-text), respectively. The nanoantenna for the measurements shown in the main-text is labeled as A-0. **e, f,** Comparison of the measured spectral response of the linear (**c**) and nonlinear (**d**) time-resolved electron oscillations between nanodevices ‘A-3’ and ‘A-0’.

**Section V. Electron Transport across Nanoantenna Junction: Simmons Tunneling Model**

Transport of electrons across the nanoantenna junction is primarily determined by the probability of electron tunneling between the apexes of the two antennas (Fig. 4a), which in turn is significantly influenced by the occupation of high-lying electronic sates above the Fermi level of Au nanoantennas ensuing absorption of photons from the laser pulse. We consider here a one-dimensional potential barrier model formed between the two apexes of the nanoantennas and the tunneling gap as schematically shown in Fig. S8. In the perturbative regime of light-matter interaction, with Keldysh parameter^2^ γ > 1, the photon-induced excitation of the electrons above the Fermi level can be modeled by an effective time-averaged Fermi population distribution function, *f*_eff_. In the case of non-perturbative light-matter interaction, where γ < 1, the potential barrier formed between the apexes of the nanoantennas can be significantly modified, this would happen at much higher local field strengths of the laser pulses at the nanoantenna junction (~ 10 V/nm).

Briefly, by utilizing the Simmons tunneling model^3,4^, the tunneling probability of electrons across the potential barrier (*U_B_*) can be expressed as;

$I(d,U_{B})\propto\int_{0}^{\infty} f_{eff}(E)\times T(E,d,U_{B})\times dE$, (16)

where *d* is the tunnelling gap between the apexes of the nanoantennas, $T(E,d,U_{B})$is the tunnelling probability of electrons, which is a function of the energy of the electron (*E*), tunnelling gap (*d*) and the applied bias in the nanoantenna junction (*U_B_*). *f_eff_ (E)* can be modelled as a parameterized sum over different Fermi population distribution functions of amplitudes$\Delta_{j}$, energy intervals *E_j_* and energy widths$\delta E_{j}$:

$f_{eff}(E)=\sum_{j=0}^{N} \frac{\Delta_{j}}{\left\{\exp(\frac{E-(U_{B}+E_{j})}{\delta E_{j}})+1\right\}}$. (17)

The energy intervals of the excited electrons (*E_j_*) and their widths ($\delta E_{j}$) can be simply considered as the multiples of the central photon energy of the laser pulse and its bandwidth; .

In the calculations, the work function of the gold was considered to be 5 eV. The local density of states on each side of the bowtie antenna was assumed to be uniform (= 1). The potential barrier formed between the two antennas was calculated by taking into consideration the image charges. The tunnelling probability of electrons was calculated within the Wentzel–Kramers–Brillouin (WKB) approximation^5^.

Upon photon absorption, electrons below the Fermi level are excited, which then create a non-equilibrium distribution of electron density from the Fermi energy up to the energy *E_j_* above the Fermi level, where *E_j_* ~ *j* * 1.6 eV. Since the energy widths ($\delta E_{j}$) of photoelectron population distribution is much smaller compared to *E_j_*, we assumed $\delta E_{j}$~ 0 in the calculations (zero temperature). That is, *f_eff_ (E)* = $\Delta_{j}$ when 0 < *E* ≤ *E_j_*, and *f_eff_ (E)* = 0 when *E > E_j_*. To simulate the bias-dependent photocurrent, the tunnelling probability of the photo-excited electrons is calculated as a function of the increasing bias, for different gap sizes.


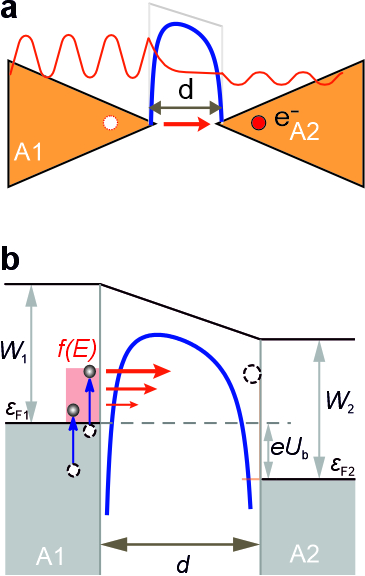


**Fig. S8 |** Schematic of the tunnelling model. **a,** Electron oscillations above the Fermi level stimulated by photon absorption at the nanoantenna apexes can lead to photo-assisted electron tunnelling across the potential barrier (blue curve). **b,** Potential-energy landscape of the nanoantenna tunnel junction at a bias voltage of *U_B_*. W_1_ and W_2_ are the work functions of the nanoantenna apexes (A1 and A2), *Ԑ*_F1_ and *Ԑ*_F2_ are the Fermi energy levels. The blue curve shows the potential barrier calculated after taking into consideration the image potential. The electrons below the Fermi level can absorb photons from the near-field and produce a non-equilibrium electron population (red rectangle).

**References**

1 Johnson, P. B. & Christy, R. W. Optical Constants of the Noble Metals. *Phys. Rev. B.* **6**, 4370 (1972).

2 Fedorov, M. V. L. V. Keldysh's "Ionization in the Field of a Strong Electromagnetic Wave" and modern physics of atomic interaction with a strong laser field. *J. Exp. Theor. Phys.* **122**, 449-455 (2016).

3 Simmons, J. G. Generalized formula for the electric tunnel effect between similar electrodes separated by a thin insulating ﬁlm. *J. Appl. Phys.* **34**, 1793–1803 (1963).

4 Schroder, B. *et al.* Controlling photocurrent channels in scanning tunneling microscopy. *New J. Phys.* **22**, 033047 (2020).

5 Wiesendanger, R. Introduction: fifteen years of spin-polarized scanning tunneling microscopy. *Microsc. Res. Tech.* **66**, 59-60 (2005).
